# Reference dosimetry using radiochromic film

**DOI:** 10.1120/jacmp.v13i6.3994

**Published:** 2012-11-08

**Authors:** Frédéric Girard, Hugo Bouchard, Frédéric Lacroix

**Affiliations:** ^1^ Département de Radio‐Oncologie Centre hospitalier de l'Université de Montréal (CHUM) Département de Physique Université de Montréal Montréal Québec Canada

**Keywords:** radiochromic film, reference dosimetry, humidity, readout temperature, noncatalytic development

## Abstract

The objectives of this study are to identify and quantify factors that influence radiochromic film dose response and to determine whether such films are suitable for reference dosimetry. The influence of several parameters that may introduce systematic dose errors when performing reference dose measurements were investigated. The effect of the film storage temperature was determined by comparing the performance of three lots of GAFCHROMIC EBT2 films stored at either 4°C or room temperature. The effect of high (>80%) or low (<20%) relative humidity was also determined. Doses measured in optimal conditions with EBT and EBT2 films were then compared with an A12 ionization chamber measurement. Intensity‐modulated radiation therapy quality controls using EBT2 films were also performed in reference dose. The results obtained using reference dose measurements were compared with those obtained using relative dose measurements. Storing the film at 4°C improves the stability of the film over time, but does not eliminate the noncatalytic film development, seen as a rise in optical density over time in the absence of radiation. Relative humidity variations ranging from 80% to 20% have a strong impact on the optical density and could introduce dose errors of up to 15% if the humidity were not controlled during the film storage period. During the scanning procedure, the film temperature influences the optical density that is measured. When controlling for these three parameters, the dose differences between EBT or EBT2 and the A12 chamber are found to be within ±4% (2σ level) over a dose range of 20–350 cGy. Our results also demonstrate the limitation of the Anisotropic Analytical Algorithm for dose calculation of highly modulated treatment plans.

PACS numbers: 87.55.Qr; 87.56.Fc

## I. INTRODUCTION

Radiochromic films offer two‐dimensional measurements, a high spatial resolution, quasi‐energy independence, and water equivalence. These characteristics render them ideal for intensity‐modulated radiation therapy (IMRT) quality control (QC), among other tasks. Their use is at present limited to relative dose measurements^(1)^ using dose normalization in order to reduce dose errors introduced by the film themselves. However, this can also potentially drown out systematic errors that are not associated to film dosimetry.^(2)^ QC tests would greatly benefit from reference dose measurements if the errors from the film can be adequately controlled. Reference dosimetry using radiochromic film would also represent an interesting alternative over the use of metal‐oxide semiconductor field‐effect transistors (MOSFETs) for *in vivo* dosimetry.

Recent work has demonstrated that under optimal conditions, absorbed dose can be determined with a film measurement uncertainty of less than 1% (one standard deviation) using EBT.^(3)^ However, EBT films are no longer commercially available, and no studies have been made to evaluate if EBT2 can be used for reference dosimetry.

The energy dependence or independence of EBT^4^, ^8^ and EBT2^6^, ^9^ was examined in detail previously. However, there are some inconsistencies in several studies concerning the level of energy dependence of radiochromic film. This was attributed to the variations of the concentrations of bromine, chlorine, and potassium in some batches of EBT and EBT2 films.^(6)^ A variation of dose rate has been shown to significantly influence the optical density of EBT in real‐time measurements.^(10)^ However, if the polymerization of the active layer is allowed to complete with time, this effect becomes negligible.^(11)^ Postirradiation development is another factor that can influence the response of EBT^(12)^ and EBT2.^9^, ^13^, ^14^ For both types of film, the optical density increases in a quasilogarithmic fashion following irradiation. Major dose errors may be made if film digitization is performed when the optical density has not yet stabilized. For this reason, it is better to perform all scans after a period of 24 to 48 hours following irradiation. Due to the light polarizing effect of the active layer of radiochromic films, there is a difference in optical density when comparing films scanned using a flat‐bed scanner with portrait versus landscape orientation.^13^, ^15^, ^18^ Unlike EBT film, EBT2 is asymmetric in its construction (i.e., the thicknesses of the polymer layers are not identical), and the measured optical density differs when films are scanned facing up or facing down on the scanner bed.^(16)^ For these reasons, it is very important to use a consistent orientation (i.e., portrait/landscape and face up/face down) when digitizing the film.

Flatbed scanners have been shown to have a nonuniform response in the regions perpendicular to the scanning direction.^17^, ^19^, ^22^ It is necessary to correct the optical densities measured to account for this artifact. This problem has been attributed to a nonuniform light production in the scanner lamp and nonuniform response of the charge‐coupled device array.^(19)^ It has also been suggested that it may be due to light scattering by the films.^(23)^ Further investigations on this effect are performed in this study.

The temperature of EBT film while scanning^17^, ^24^, ^25^ and the level of hydration of its active layer^(25)^ are known to affect optical density readings. These factors were characterized in detail for EBT2 in this study. Although EBT2 films are far less susceptible to ambient light than EBT films,^11^, ^13^ there is an intrinsic development of radiochromic films with time that cannot be avoided, even in the absence of radiation. This noncatalytic polymerization of the active molecules or “autodevelopment” affects the long‐term stability of the optical density of the film. As a result, calibration curves will not remain stable over time. The stability of calibration curves, including the impact of storage temperature, was evaluated in this study.

The aim of this study is to perform reference dose measurements with radiochromic film (EBT2) using an optimal measurement protocol and to determine the associated uncertainties. Multiple parameters may influence the film response and introduce systematic dose errors. Some of these parameters and their impact on EBT2 response have been studied previously.^6^, ^9^, ^13^, ^14^, ^16^ Others, such as humidity, readout temperature, and noncatalytic development, have not and are investigated for the first time for EBT2 film. Because EBT2 films are very different from their predecessor (for example, a dye was added to the active layer of the film and the binding agent of the film was altered), it is necessary to study these films in detail even though some results are available for EBT film. Based on these results, optimal conditions for reference dosimetry were elaborated and tested for IMRT QC.

## II. MATERIALS AND METHODS

### A. Film preparation and irradiation procedure

Three lots of EBT2 film (International Specialty Products Corporation, Wayne, NJ) were used: lots F10070901A, F0813092B and F04090903. In addition, one lot of EBT film (48022‐08I) was used for comparison in some experiments. All experiments with EBT2 film were carried out within the company‐recommended lifetime of the film. However, this wasn't the case for EBT films, since they were no longer available commercially and older lots needed to be used. This only influences the intrinsic optical density of the film prior to irradiation which is slightly higher than usual for those films.

Randomly selected pieces of film of $2.5\times 2.5\;{\text{cm}}^{2}$ were placed at an SAD of 100 cm and a depth of 2 cm inside a Plastic Water DT phantom (CIRS Inc., Norfolk, VA) of $30\times 30{\;\text{cm}}^{2}$. Each piece of film was irradiated using a Varian Clinac 21EX (Varian Medical Systems, Palo Alto, CA) in a $10\times 10{\;\text{cm}}^{2}$ field and a 6 MV photon beam. For calibration purposes, at least 15 pieces of film irradiated at various doses ranging between 0 and 800 cGy were used. The linac output was measured using an Exradin A12 ionization chamber (Standard Imaging, Middleton, WI) calibrated at the National Research Council of Canada (Ottawa, Canada). The chamber was placed at the isocenter and a depth of 10 cm, as recommended in the TG‐51 protocol,^(26)^ inside a Plastic Water DT phantom. MU values were converted to absorbed dose, using the absorbed dose‐to‐water calibration factor determined with the A12 ionization chamber measurements. For reference dosimetry with EBT and EBT2, doses were calculated using an independent calibration curve and compared to doses obtained using the ionization chamber correction factor. In this paper, the expression “reference dosimetry” or “reference dose measurements” is used to refer to a measure of the dose with the film that is counter‐calibrated using an ionization chamber. This dose value is not normalized by another value, as they would for relative measurements.

### B. Scanning protocol and homogeneity correction

Films were scanned using the protocol described by Bouchard et al.^(3)^ They were scanned five times prior to irradiation and 38 to 42 hours after the irradiation using an Epson 10000 XL flatbed scanner (US Epson, Long Beach, CA) in reflection mode. Net optical density was obtained by subtracting the optical densities of the film measured before irradiation to the one measured after irradiation. The orientation of the film on the scanner bed was kept constant. If the scanner remained inactive for more than 20 minutes, five blank scans were performed to warm up the lamp and the scanner bed. Film temperature was monitored using an infrared thermometer (Oakton Inc., Vernon Hills, IL). Unless indicated otherwise, temperature variations on the scanner bed were less than 1°C for all experiments. All the films were digitized in 48‐bits RGB format at a resolution of 150 dpi and saved in uncompressed TIFF format. The images were processed using an open source program written in MATLAB 7.4 (The MathWorks, Natick, MA) called Gafgui.^(27)^ Film analysis was performed on the red channel component of the image. It should be noted that at the time of measurements using EBT2, the film manufacturer recommended using the red channel component of the image normalized to the blue channel component in order to eliminate the effect of film thickness variations. However, despite the fact that errors due to the thickness of the active layer were correlated for the red and blue channels, it was found that other components, such as the intrinsic noise from the scanner readings, nevertheless increased the overall uncertainty. It was found that using only the red component was a better choice over using the red and blue correction, as suggested in the ISP white paper.^(11)^

All scanned images were corrected for the inhomogeneity of the scanner response using a method inspired by Saur and Frengen.^(22)^ However, instead of using small square regions of film, several bands of $20.32\times 2\;{\text{cm}}^{2}$ were uniformly exposed using a 9 MeV electron beam. The bands were placed inside a $25\times 25{\;\text{cm}}^{2}$ field diagonally (in order to exploit the most uniform region) at an SSD of 100 cm and a depth of 2.1 cm inside the same phantom used for the calibration procedure. Twelve bands were irradiated to cover the optical density range used for the experiments. The film bands were scanned five times together at the center of the scanner bed, and a homogeneity correction matrix was constructed using multiple 8‐degree polynomial functions. In order to cover a broader range of the scanner, the bands were translated to the rightmost and leftmost region of the scanner bed and digitized independently. Both sets of images were averaged and recombined numerically using the right and left half of the corresponding translated image. This final image was used to create a homogeneity correction matrix that covers the entire scanner bed. A homogeneity correction matrix was also created using a transmission step wedge (Stouffer Industries Inc., Mishawaka, IN, USA) instead of radiochromic film, following the same procedure.

### C. Temperature and humidity control

EBT and EBT2 film were stored in different humidity conditions. Temperature and relative humidity were monitored using a Track‐It data logger (Monarch Instruments, Amherst, NH, USA). In order to control the humidity level, the films were kept inside a sealed container in the presence of desiccant drierite (W.A. Hammond DRIERITE Co. LTD, Xenia, OH, USA) or a water bath. The relative humidity was maintained under 20% or above 80%, respectively. Films were stored in these conditions for a minimum period of two weeks prior to the experiments in order to equilibrate the level of hydration of the active layer with the environmental humidity. Each piece was cut from the same sheet of film. In addition, the two sets of films were irradiated at the same time.

**Figure 1:**
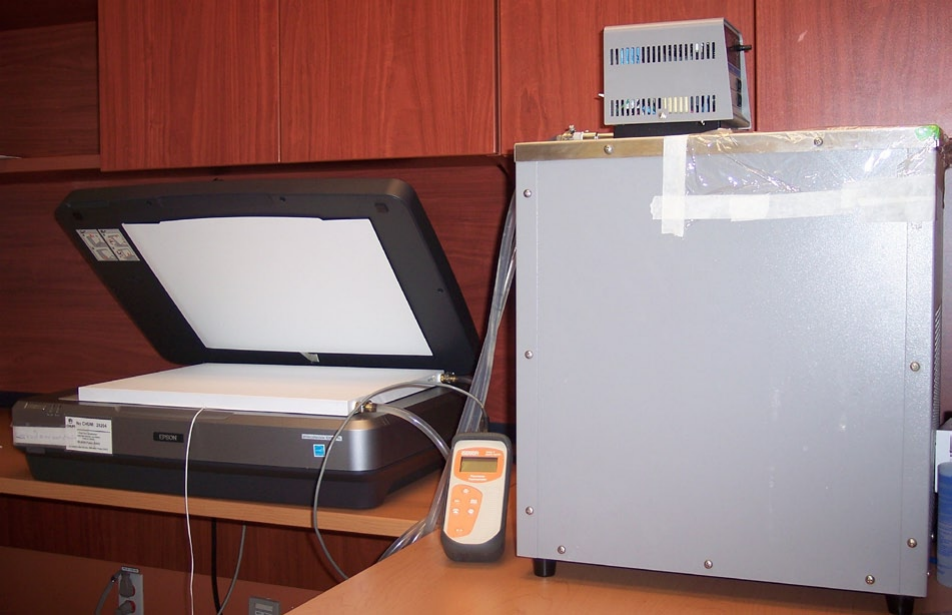
Scanner bed temperature experimental setup: water coming from the heating/refrigerating pump to the right is circulated inside a hollow slab placed over the reflector, which is over the scanner bed and the film. A small surface thermistor is placed at the slab–reflector interface.

It is known that film optical density increases with time even if the polymerization reaction is not catalyzed by ultraviolet light or ionizing radiation. To evaluate if it is possible to stop or slow down this reaction and thus to stabilize the calibration curves, the films were kept inside a refrigerator (~ 4°C). Film temperature is also known to influence the optical density reading while scanning. This variation was studied for standard room temperatures (18°C to 30°C). The temperature of the scanner bed and the film was controlled using the apparatus shown in Fig. 1. Pieces of film were placed at the center of the scanner bed where the homogeneity correction is negligible. A thin layer of reflector material was placed over the film. A polystyrene slab was placed over the reflector inside which water was circulating. Water temperature was controlled using a heating/refrigerating pump (PolyScience, Niles, IL, USA). A small surface thermistor probe (Oakton Instruments, Vernon Hills, IL, USA) was placed at the reflector‐slab interface to monitor the temperature. During the determination of temperature and humidity effects on film, digitization was performed only once to prevent unwanted warming of the scanner bed. Temperature was increased at intervals of 1°C, with 15 minutes between readings in order to allow sufficient time to reach thermal equilibrium.

### D. IMRT QC

The IMRT QC procedure consisted of generating a verification plan of a patient's treatment plan using a Plastic Water DT phantom. The phantom was composed of two 5 cm slabs ($30\;\times \;30\;{\text{cm}}^{2}$), a 2 cm slab in which a Farmer ionization chamber (PTW, Freiburg Germany) was placed, and a 4 cm slab. An EBT2 film was placed between the 2 cm and 4 cm slabs. The field setup of four randomly selected treatment plans was used to irradiate the phantom, the film and the ionization chamber. The phantom was imaged using a Big Bore (Philips, The Netherlands) CT scan and imported into the treatment planning system Eclipse 8.9 (Varian Medical Systems, Palo Alto, CA). Dose was calculated using the Anisotropic Analytical Algorithm without heterogeneity corrections and a grid size of 1 mm resolution. A coronal 2D plane ($30\;\times \;30\;{\text{cm}}^{2}$) located at the film's position was selected for each plan and exported to be compared to the measured film using the geometric gamma approach.^(28)^ The analysis was performed with a homemade MATLAB program functioning in reference or relative mode using a 4% dose threshold and 4 mm distance threshold, or 3% dose and 3 mm distance, or 2% dose and 2 mm distance. All exported dose distributions were corrected to account for the variation of the linac output on the day of measurement. Doses below 10% of the maximum value were excluded from the comparison. IMRT QC of the same plans was also performed using the ${\text{Delta}}^{4}$ diode array (ScandiDos, Uppsala, Sweden).

The calculated dose distributions were compared with dose to film measurements. In order to eliminate the influence of the readout temperature, humidity, and autodevelopment, pieces of film used for reference dosimetry and the ones used for the calibration curve were irradiated on the same day, and they were also scanned simultaneously. All the films used were irradiated independently. A large number of calibration points were needed to achieve high accuracy.^(3)^ Specifically for reference dosimetry, a total of 45 calibration points were used to cover a dose range of 0 to 440 cGy.

The dose distribution obtained from the film was adjusted proportionally to the daily linac output. The absorbed dose was determined in Plastic Water DT, using an A12 ionization chamber. Using the known composition of Plastic Water DT, the chamber calibration coefficient^(26)^ (e.g., $N_{D,w}$) was corrected for stopping‐power ratio plastic water to water in order to estimate the calibration coefficient in plastic water (e.g., $N_{D,\text{pw}}$). The measured absorbed dose in plastic water was then corrected by the ratio of mass energy‐absorption coefficient ($\mu_{\text{en}}/\rho$) of water to Plastic Water DT, while the fluence attenuation correction was found to be negligible. For the 6 MV beam, the overall absorbed dose correction was estimated to be 0.9987, which is very close to unity.

The measured dose distributions were subsequently corrected in the same fashion, but this time using the Farmer chamber measurements. The Farmer chamber measurement gives a gross estimate of the systematic error either introduced during the delivery or due to the limitations of the TPS in calculating the dose accurately. However, it is important to also consider that ionization chambers are subject to errors when measuring IMRT fields,^(29)^ even when placed in low‐gradient regions within the plan, such as was the case here. The corrections were carried out under the assumption that the measurements performed with the Farmer chamber were done under ideal conditions.

### E. Film characterization and uncertainty analysis

Film characterization to absorbed dose and uncertainties was performed as described by Bouchard et al.^(3)^ Film dose‐response was characterized using a mathematical function in respect to the convergence of the uncertainty estimate, as well as the unbiased nature of the residuals. As for the uncertainty analysis, the method considers mainly the statistical nature of the calibration process and measurements, and accounts for statistical correlations in the estimation of the uncertainty. In this study, the impact of temperature or humidity variations and autodevelopment was examined. Such variations are likely to introduce systematic dose errors. The magnitude of these errors was measured by calculating two calibration curves, determined under various conditions (i.e., one curve determined at high humidity and the other at low humidity). The difference between absorbed doses estimated with both calibration curves was interpreted as the estimate of the error. The comparison was limited to a dose range of clinical interest (20 to 350 cGy). The lowest threshold equivalent to 10% of 2 Gy was chosen in order to exclude irrelevant low‐dose regions from the analysis. The highest threshold was selected in order to include the maximum doses observed in IMRT QC verification plans.

## III. RESULTS

Each experiment was carried out with all three lots of EBT2 film. Results presented in figures are from one lot only, but they are representative of all three lots of films.

### A. Homogeneity correction

**Figure 2:**
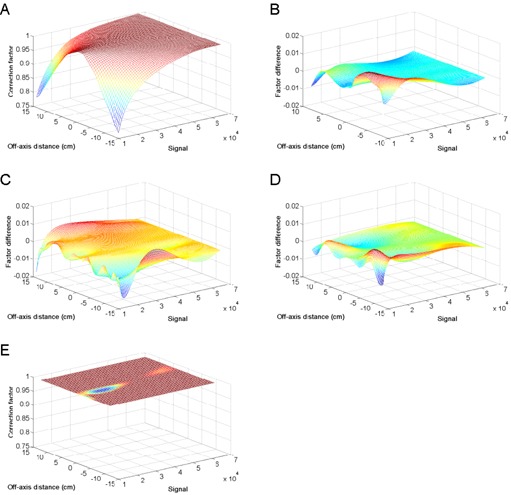
Homogeneity correction: an extended correction matrix of EBT2 films lot F10070901A (A) and its difference with: the regular centered matrix (B), EBT2 films lot F0813092B (C), and EBT films lot 48022‐08I (D). A correction matrix (E) obtained with an optical density standard.

The response of flatbed scanners has been shown to vary in the direction parallel to the lamp.^17^, ^19^, ^22^ A homogeneity correction matrix can be constructed from the images of uniformly exposed films^(22)^ by calculating the pixel value differences relative to the center of the scanner bed in order to correct for this variation. This correction matrix is limited to the region where the film bands are present, and is about 20 cm wide (the regular matrix), but can be extended by translating these bands to the left and right side to obtain a 30 cm wide matrix (large matrix). Once both images have been recombined, the correction matrix will cover the desired area of the scanner. (Figure 2a) presents the homogeneity correction matrix calculated in this fashion for a length of approximately 28 cm. The difference between this large matrix and a regular matrix was calculated over the common region ((Fig. 2b). The root mean square differences were of the same order of magnitude of the uncertainties in optical density measurements, which are typically 0.002 (in optical density). However, the differences between both matrices were slightly more pronounced as the distance from the central region increases. This is attributed to the unreliability of the fitted function at the edge of the matrix. For this reason, it is preferable to create a large correction matrix and to use a limited region of 20 cm wide, the length of a complete EBT2 sheet.

Correction matrices of three lots of EBT2 film were compared. An example is shown in (Fig. 2c). The difference between correction matrices created from EBT and EBT2 film is also examined ((Fig. 2d). As is observed for a single film lot ((Fig. 2b), the differences are statistically insignificant for the major part of the matrices, but are significant at the edge of the matrices ((Figs. 2c) and (d)). It can therefore be concluded that the homogeneity correction is not dependent on the type or lot of the films. This also confirms that when using large correction matrices, it is preferable to limit their use to the central region where variations are smaller.

A correction matrix was also created using a transmission step wedge ((Fig. 2e). This was composed of a single layer of photographic film. The wedge covered a range of optical densities that included that of EBT2 films for a dose range of 0 to 800 cGy, yet there were no inhomogeneities present and therefore no correction was necessary for this wedge. This clearly indicates that the inhomogeneities observed in EBT and EBT2 were not solely due to the scanner, but to an interaction between the scanner light and radiochromic films.

### B. Humidity

**Figure 3:**
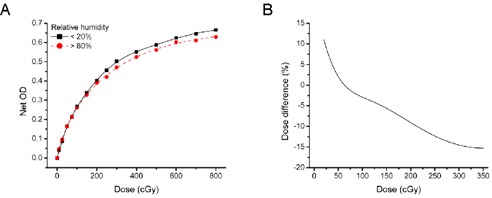
Influence of relative humidity on radiochromic film response: the response of EBT2 film using 15 calibration points (A) for high and low humidity level. The curves are the calibration function fitted to the data points represented by symbols. The dose differences (B) obtained from the estimated value from both calibration functions.

The response of EBT2 films varied considerably with the humidity level ((Fig. 3a). The net optical density of films stored at low humidity was systematically higher than the net OD of film kept at a high humidity for optical densities above 0.2. Storing the film at low humidity appeared to increase the sensitivity of the film. Calibration curves were created from both high‐ and low‐humidity films and they were compared by calculating the difference between the dose values obtained ((Fig. 3b). If one would use an incorrect calibration curve, for example a calibration curve constructed with pieces of film stored at high humidity (>80%) while the measurements were performed with pieces of film stored at low humidity (>25%), the resulting absorbed dose errors could reach up to ~ 15%. Similar results were obtained with each lot of EBT2 film, with some variation of the maximum dose error observed (from 15% to 20%). This is potentially the single most important source of error when using radiochromic films. Great care should be taken to perform calibrations and measurements under consistent humidity conditions. Preferably, the film should be stored at a low humidity level to increase its sensitivity.

### C. Readout temperature

**Figure 4:**
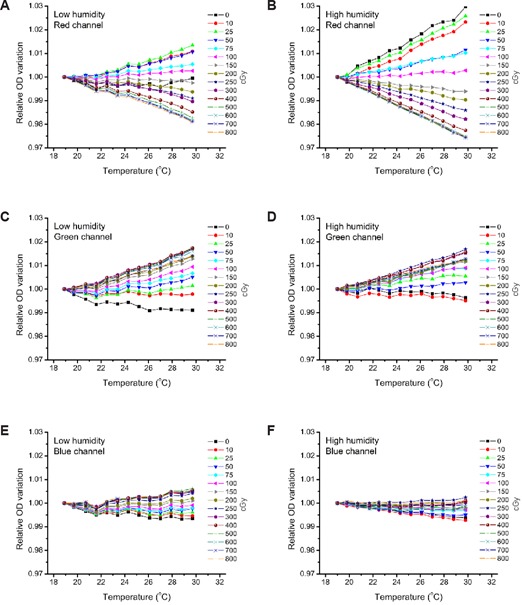
Influence of scanner bed temperature on optical density readings: results from the red (A, D), green (B, E), and blue (C, F) channel for film stored at relative humidity inferior to 20% (A, B, C) or superior to 80% (D, E, F). Raw optical density readings are normalized relative to the lowest temperature value.

The temperature of the film when scanning is known to influence the optical density readings of EBT films.^17^, ^24^, ^25^ The impact of this factor was evaluated for EBT2 using pieces of film that were previously stored under low humidity conditions (<20%). The experiments were performed independently with three lots of film. Representative results for the red, green, and blue channels are shown in (Figs. 4a), (b), and (c), respectively. Each point has been normalized based on the raw optical density value at the lowest temperature (19°C). The relative change of optical densities was more important for the red channel than for the other channels. Variations observed depended on the dose received by the film. For the red channel, the variations were more important for the lowest and highest dose values and are almost negligible for doses around 150 cGy. Low‐dose value curves were also more variable than others due to their initial low optical density and the relative representation used. A maximum rate of optical density change of ±0.15% per °C was observed for the red channel. These results indicate that the dependence on temperature was mostly due to the active layer, since the variations observed for the green and blue channel were less important than the ones for the red channel. Similar experiments were also performed under high humidity conditions (>80%). The general behavior of the film with temperature was comparable to low humidity results for the red ((Fig. 4d), green ((Fig. 4e), and blue ((Fig. 4f) channels. However, the rate of change of optical density was more pronounced for the red channel, with a maximum rate of ±0.25% per °C.

A variation of ±1°C had only a minor impact on the calculated absorbed dose. Differences were within ±0.5% for all three EBT2 lots tested at the dose range (0 to 800 cGy) measured. For a temperature variation of ±2°C, the differences were mostly within ±0.5%, but sometimes reached up to 1% for some lots and for limited dose points at a dose range of 20 to 350 cGy. Errors became more significant beyond this range. For optimal accuracy, the temperature should be kept to within ±1°C of the temperature measured when constructing the calibration curve. This can be easily achieved by using an experimental setup similar to the one shown on Fig. 1. If such a system is not available, the scanner can be used to warm up the film and the scanner bed to a reference temperature point which should be used for all scans. Temperature variations before and after five scans are observed to be within ± 0.5°C.

### D. Noncatalytic development

**Figure 5:**
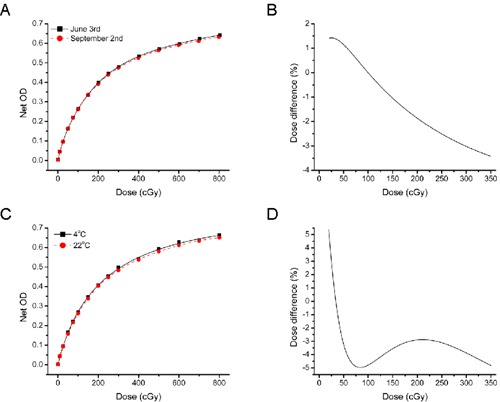
Autodevelopment of EBT2 films: the response of EBT2 before and after a period of 3 months (A) and the dose differences between both curves (B). The response of EBT2 films stored at room temperature and inside a refrigerator for a period of 8 months (C) and their dose difference (D). 15 points were used for each calibration.

The autodevelopment of radiochromic film represents an important obstacle to reference dosimetry as it results in a shifting calibration curve. Even if the films are kept in the dark, the optical density of the unexposed film still increases with time ((Fig. 5a). There was a notable difference between the calibration curve performed in June with the one in September, and net optical densities for high‐dose values were clearly reduced after this three‐month period. Ionizing and ultraviolet radiations are catalysts in the polymerization of the active molecule, but their presence is not mandatory. This chemical reaction can still occur in the absence of radiation, albeit at a much lower rate.

Films were stored in a refrigerator, as recommended by the manufacturer.^(11)^ Temperature and humidity were monitored. In this case, different calibration curves were performed at different times. Hence, the dose received was verified using an ionization chamber, as described in Methods & Materials Section A, above, in order to properly compare each calibration curve. A three‐month difference between both calibration curves ((Fig. 5a) can introduce important dose differences that may reach values of up to 3.5% ((Fig. 5b). This effect varied from one lot to the other, and the maximum dose difference varied from 1% to 4%. The storage temperature had a strong impact on the autodevelopment of the film. After a storage period of eight months, two calibrations curves were created: one with films stored at room temperature and the other with refrigerated films ((Fig. 5c). The net optical densities of films stored at room temperature were systematically lower than the refrigerated ones (with the exception of the lowest dose points for one lot). This results in a maximum dose error that can reach 5% ((Fig. 5d) or more depending on the lot (up to 8%). These results demonstrate that autodevelopment can be slowed, but not completely stopped by refrigerating the film at 4°C.

### E. reference dosimetry

**Figure 6:**
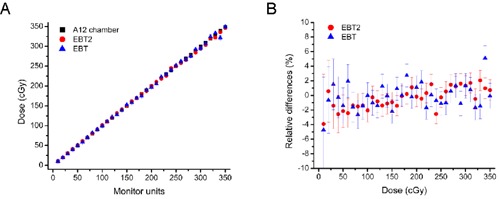
Reference dose measurements: the absorbed dose values measured with the Exradin A12 chamber as well as EBT and EBT2 films (A). A total of 34 measurements are used to compare absorbed dose predicted with EBT and EBT2 film with absorbed dose determined with the ionization chamber (B).

Results obtained with EBT and EBT2 films were compared to the dose measured with the A12 ionization chamber over a dose range of clinical interest ((Fig. 6a). For both EBT and EBT2 film, the dose values measured were in good agreement with the ionization chamber. This was also illustrated by looking at the distribution of the differences between the chamber and the film ((fig. 6b). Over a dose range of 20 to 350 cGy, 75% of the points were within 1 standard deviation, and 99% were within 2 standard deviations. Since the expected values for a Gaussian distribution are, respectively, 68% and 96%, this demonstrates that the analysis of uncertainties presented previously is accurate.^(3)^

**Figure 7:**
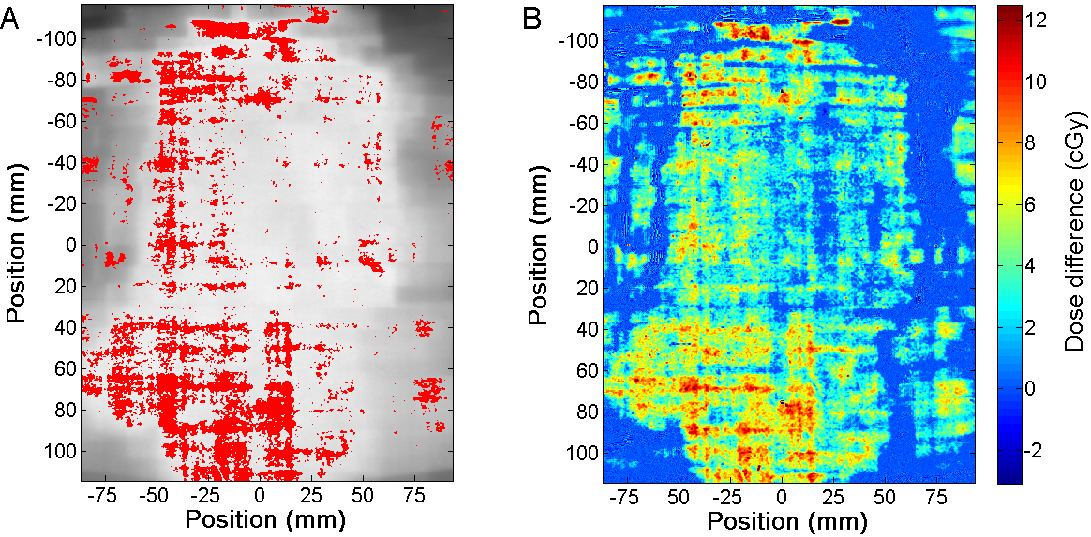
Gamma test (A) and dose difference (B) results from the abdominal treatment plan. A 3% reference dose and 3 mm distance thresholds were used for the calculation of the gamma index. Pixels failing the gamma test are shown in red. Dose distribution was only corrected for the daily output of the accelerator.

IMRT QC test were also performed for reference dose and compared to relative dose measurements (Table 1). An example of the gamma test results for the abdominal plan is presented in (Fig. 7a) for a 3% reference dose and 3 mm distance thresholds. Dose differences between the measured and planned distributions have also been calculated ((Fig. 7b). There are various criteria which determine if a plan is acceptable or not. For the prostate and head‐and‐neck plans, the gamma values are in good agreement in reference and relative mode, but there were considerably fewer pixels that pass the gamma test in reference dose for all three groups of thresholds values for the abdominal plan. The control points of this plan are composed mostly of small openings and the total monitor units are higher than for the head and neck and prostate plans for the same fraction dose. This indicates that the abdominal plan is more modulated than the head and neck and prostate plan.

**Table 1:** IMRT QC gamma test results.

		*Treatment Plan (%)*			
		*Head and Neck*	*Prostate*	*Abdominal*	*Pelvis*
	2% 2mm^b^	94.55^c^	99.16	88.67	98.24
Relative^a^	3% 3mm	99.88	99.95	98.67	99.72
	4% 4mm	99.98	99.97	99.88	99.90
	2% 2mm	94.91	98.11	52.71	83.02
Reference^d^	3% 3mm	99.90	99.90	84.28	94.41
	4% 4mm	99.98	99.97	97.37	99.18
${\text{Delta}}^{4}$ ^e^	3% 3mm	94.60	100.00	83.7	100.00

^a^Dose distributions are normalized based on the maximum

^b^dose and distance to agreement tolerance, respectively

^c^percent of pixels with gamma value ≤1

^d^dose distributions are left unnormalized

^e^performed in reference dose.

In all cases, the IMRT absorbed dose measured using the ionization chamber was close to the calculated value of the TPS. The differences were of ‐1% (head and neck), 0.8% (prostate), ‐1.1% (abdominal), and ‐0.8% (pelvis). These quantities were statistically higher than the estimated uncertainty of 0.3% based on the work from Chung et al.^(30)^ using a plan‐class–specific reference field for a typical head‐and‐neck IMRT plan. The average dose differences between both dose distributions were also very small (‐0.28, 0.18, ‐2.65, and ‐1.35 cGy, respectively), and follow a similar pattern to the result from the ionization chamber. Results from the Delta^4^ system in reference dose were in good agreement with the corresponding values from EBT2 films (Table 1).

## IV. DISCUSSION

The results shown on (Fig. 2e) indicate that the inhomogeneities observed when scanning with radiochromic film were not attributable to inadequate uniformity of the lamp or response of the charge‐coupled device array as was speculated.^(19)^ The inhomogeneities observed were attributable to the film and may be caused by light scattering.^(23)^ Since the charge‐coupled device only measures light that is perpendicularly incident to its surface, scattering would affect the amount of light that is received by it. However, this phenomenon appears only for the red color channel, and not for the green and blue ones (data not shown). This is the opposite of Rayleigh scattering, which is dominant at low wavelengths (i.e., in the blue region of the spectrum). The results could be explained by a molecular fluorescence phenomenon in which light was absorbed by the polymers causing them to enter an excited state. Relaxation through intermediate or directly to the ground state emits light of lower frequency.^(31)^ If this hypothesis is correct, the emission spectrum during relaxation must be in the red channel of the scanner, and the fluorescence must be resonant or quasiresonant in order to account for our observation. As opposed to atoms, the energy spectrum of the molecules is not solely composed of electron energetic states but a coupling of energetic, vibration, and rotational states (rovibronic coupling). These rovibronic energy states are closer together, and less energy may be lost to the internal conversions during the fluorescence process. The amount of light emitted in this fashion is proportional to the concentration of polymers formed and may explain why the inhomogeneities are more pronounced at high optical density values.

Little research has been performed to evaluate the impact of humidity on the most recent generation of radiochromic film. Rink et al.^(25)^ have observed a shift of the main peak in the absorbance spectrum with humidity in EBT. It is likely that a similar modification occurs in EBT2, since the active layer of both films is composed of the lithium salt of pentacosa‐10,12‐diynoic acid. They also observed that rehydration of an already desiccated film did not reverse the spectrum to its original form. It was concluded that water strongly influences the structure of the active molecule which, in turn, influences its absorbance spectrum. Our results are in agreement with this conclusion.

Humidity is reported to influence the dosimetry of radiochromic film in the American Association of Physicists in Medicine Task Group 55 report.^(1)^ The impact of humidity on an older generation of film (HD‐810) is less than ±2% for a humidity range of 6% to 94%.^32^, ^33^ However, HD‐810 films are far less sensitive to radiation than EBT or EBT2 films, and the composition of the active layer is slightly different. Temperature readout effects similar to what we have shown (Fig. 4) were demonstrated by Buchauer et al.^(24)^ and Lynch et al.^(17)^ with EBT film. A strict comparison is not possible, however, since the humidity conditions in both studies are unknown. This could also explain the slight variations observed between both reports.

Except for one point, all dose measurements were within ±3% (2σ) of the results obtained with the ionization chamber (Fig. 6). These results compare favorably to MOSFET performance, for example, and they open the door to reference dosimetry using radiochromic films for applications such as *in vivo* dosimetry. Three of the four IMRT QC tests showed excellent results in reference dose at 3%/3 mm (Table 1). The fourth plan, the abdominal case, is composed of several small fields which may not be appropriately modeled by the treatment planning system.

The source of discrepancy between reference dose measurements and the calculated dose distribution may also come from systematic errors introduced by the plan delivery independently of radiochromic film manipulation. The total output values measured by the Farmer ionization chamber may be used as a gross approximation of the systematic error introduced in each plan, as they are shown to be statistically higher than results previously reported by Chung et al.^(30)^ Dose distributions measured by EBT2 films were corrected based on the chamber values, and the gamma index was recalculated (Table 2). For every plan and every tolerance value, the results of the gamma test using the corrected dose distributions were either highly improved or identical to the original values. Delta^4^ results are in accordance with EBT2 measurements. These results indicate that the precision of the Anisotropic Analytical Algorithm when calculating complex dose distributions might be the limiting factor. It was recently demonstrated that version 8 of the Anisotropic Analytical Algorithm and the use of a larger dose grid resolution may not adequately calculate doses for plans composed of a large combination of small fields. ^(34)^ The reported dose deviation while using version 8 and a dose grid resolution of 1 mm (the same conditions used in this study) are of 3% and 6% for a $1\times 1{\;\text{cm}}^{2}$ symmetrical field in polystyrene and cork, respectively. These results further demonstrate that radiochromic film may be used to identify otherwise undetected systematic errors.

**Table 2:** Corrected gamma test results.^a^

		*Treatment Plan (%)*			
		*Head and Neck*	*Prostate*	*Abdominal*	*Pelvis*
Reference^d^	2% 2mm^b^	98.05^c^	99.16	81.61	91.31
	3% 3mm	99.97	99.94	97.33	98.40
	4% 4mm	99.98	99.97	99.73	99.90

^a^Dose distribution measured with EBT2 films and compared with corrected plan based on the IMRT output factor measurement using the Farmer chamber

^b^dose and distance to agreement tolerance, respectively

^c^percent of pixels with gamma value ≤1

^d^dose distributions normalized to ion chamber results.

Due to the short lifespan of the calibration curve for reference dosimetry, which we estimate to be no more than a week, the use of radiochromic film on a routine basis for IMRT QC would be time‐consuming due to the need to construct a calibration curve for every measurement run. For this reason, reference dosimetry using radiochromic film may be more appropriate for specialized tasks, such as for the commissioning of new equipment, where its spatial resolution is unmatched by the array dosimeters available on the market today.

Recently, the manufacturer of GAFCHROMIC film released a new generation of film called EBT3. The active layer of these new films is the same as for previous generations, and they are symmetrical in construction, like EBT film. In addition, the film features an anti‐Newton ring coating. Since the influence of humidity, temperature, and noncatalytic development is related to the active layer of the film, it is most likely that EBT3 will be influenced as well by these factors, and similar precautions to those presented in this study should be taken when using EBT3 films.

## V. CONCLUSIONS

In order to perform accurate dose measurements using radiochromic films, it is advisable to take the following precautions:

i) Keep the orientation (i.e., landscape/portrait and face up/down) of the film on the scanner bed consistent;
ii) Characterize film versus absorbed dose using the same beam energy as used for measurements;
iii) Keep postirradiation wait time constant and long enough to allow film to reach stability;
iv) Correct scanner images for the inhomogeneities of the scanner response;
v) Ensure that the film temperature when scanning is kept within ±1°C of the recorded temperature during the calibration; and
vi) Store film at a constant relative humidity index and at low temperature in order to reduce the autodevelopment.

Under these optimal conditions, it is possible to perform reference dose measurements using radiochromic film with uncertainties of ±2% (1σ). This precision is sufficient to allow the use of radiochromic film in reference mode for *in vivo* measurements, IMRT QC, and commissioning tasks.

## ACKNOWLEDGMENTS

We would like to thank Professor Jean‐Louis Schwartz, Université de Montréal, for the loan of the heating/refrigerating pump. We would also like to thank Jean‐Philippe Gariépy for performing the Delta^4^ measurements. This work was supported by grant 357402 from the Natural Sciences and Engineering Research Council of Canada. Frédéric Girard gratefully acknowledges funding via the Programme de maîtrise en physique médicale from the Ministère de la santé et des services sociaux du Québec.
